# The role of *Bordetella pertussis* in the development of multiple sclerosis

**DOI:** 10.1186/s12883-022-02606-4

**Published:** 2022-03-01

**Authors:** Mohammad Mahdi Majzoobi, Mohammad Reza Macvandi, Hamidreza Ghasemi Basir, Zahra Sanaei, Shahir Mazaheri, Maryam Afza, Mohammad Reza Arabestani

**Affiliations:** 1 Department of Infectious Diseases, School of Medicine, Hamadan University of Medical Sciences, Hamadan, Iran; 2 Brucellosis Research Center, Hamadan University of Medical Sciences, Hamadan, Iran; 3 Department of Pathology, School of Medicine, Hamadan University of Medical Sciences, Hamadan, Iran; 4 Department of Community Medicine, School of Medicine, Hamadan University of Medical Sciences, Hamadan, Iran; 5 Department of Neurology, School of Medicine, Hamadan University of Medical Sciences, Hamadan, Iran; 6 Department of Microbiology, School of Medicine, Hamadan University of Medical Sciences, Hamadan, Iran

**Keywords:** Multiple sclerosis, *B. pertussis*, Polymerase chain reaction, Infectious agents, Serologic test

## Abstract

**Background:**

Multiple sclerosis (MS) is one of the most common neurological disorders which main cause is not identified yet. Some studies mentioned the possible role of infectious agents such as chlamydia pneumonia, mycoplasma and also, *B. pertussis* via asymptomatic nasopharyngeal colonization. The current study aimed to investigate and compared the serum level of *B. pertussis* antibody and the rate of nasopharyngeal colonization by this pathogen in subjects with and without MS.

**Methods:**

In this case-control study, 109 patients with MS and 114 subjects without MS referred to Sina Hospital in Hamadan in 2019 are studied and compared in terms of serum titer of *B. pertussis* antibody and nasopharyngeal colonization by this bacterium. Colonization was evaluated using culture and real-time PCR techniques. Data were analyzed using SPSS version 16 with a 95% confidence interval.

**Results:**

The serum titer of *B. pertussis* antibody in case and control groups was 37.8 and 35.1%, respectively (*P* = 0.74). Culture and real-time PCR techniques revealed no case of nasopharyngeal colonization by *B. pertussis*.

**Conclusion:**

There was no difference between *B. pertussis* antibody titer and the rate of nasopharyngeal colonization between both MS patients and the healthy control group. Therefore, it seems that probably *B. pertussis* has not a role in MS development.

## Background

Multiple sclerosis (MS) is one of the most common neurological disorders all around the world, corresponding to 100 per 100,000 people in Europe and North America to 2 per 100,000 people in areas close to the Earth’s Equator. It is estimated that 2.5 million people live with MS globally. In Iran, the prevalence of MS ranges from 5.3 to 74.28 per 100,000 people [1–4]. MS is more common in young people, and early symptoms generally begin before the age of 55. The maximum incidence of the disease is in the ages of 20 to 40 years and the genetic factors have a key role in its development [5–7]. The unequal risk of MS development in monozygotic twins indicates in addition to genetic predisposition, environmental factors are important for MS development [8, 9].

Although some researchers have provided evidence that infectious agents play a significant role in the etiology of MS, it should be noted that to date, no specific virus or bacteria alone is introduced as the cause of MS development. It is not yet clear exactly that infectious agents play a direct role in the etiology of MS or these factors only facilitate and intensify the disease [7].

The most important evidence that indicates a significant association between MS and infectious agents is the fact that 90% of MS patients have an abnormally elevated titer of immunoglobulin G in their cerebrospinal fluid (CSF) [10]. In contrast, there is a hypothesis that high concentrations of IgG in CSF of MS patients may be the cause of the disease [11]. Some evidence indicating that asymptomatic colonization of *B. pertussis* can be the cause of MS. *B. pertussis* colonization is common in children, youth, and adults [12, 13].

The high risk of MS in the U.S. is reported to be indirectly associated with pertussis vaccination. It is believed that pertussis vaccination can reduce the incidence of acute and clinical forms of the disease, but it does not exert a mucosal protective effect [13–15]. Therefore, it is postulated that cases with subclinical pertussis are at elevated risk of MS [14, 15]. The human nasopharynx can be colonized with bacteria and pathogens such as *B. pertussis* [16]. But no chronic carrier in humans is reported [17]. Rubin et al. [18] reported that subclinical colonization of *B. pertussis* in nasopharyngeal of vaccinated individuals may be associated with MS development. In animal models, also *Bordetella pertussis* has experimentally caused MS.

Kurtzke et al. published one of the most important epidemiological reports on MS in 1993. Based on the MS epidemic in the Faroe Islands during World War II and immediately after the war, they concluded that MS is a late consequence of a particular unknown infection during adolescence and youth [19]. A study showed that 95.47% of MS patients were positive for anti-Bordetella antibodies [20]. Although an association is postulated between nasopharyngeal colonization and MS, no study on isolation of this bacterium from nasopharyngeal was found. Therefore, this case-control study was conducted to investigate the serum level of antibody and the rate of *B. pertussis* nasopharyngeal colonization in patients with MS and subjects without MS.

## Method

This case-control study was conducted on subjects with and without MS, referred to Sina Hospital in Hamadan, west of Iran, in 2018-2019. After obtaining informed consent, participants were enrolled in the study. The cases were selected using convenience and consecutive sampling techniques among eligible patients. The sample size was calculated as 124 subjects, following the study by Sherkat et al. [21]. (Prevalence of positive *B. pertussis* antibody in adults referred to Isfahan medical centers), with an alpha of 0.5, 0.06 error, and a prevalence of 0.12.

Out of 109 patients with MS were compared with 114 participants without MS. MS patients were enrolled according to clinical symptoms, laboratory findings (increased IgG CSF and oligoclonal band in CSF), and imaging evidence provided by MRI, which all were confirmed by a neurologist. Subjects of the control group (i.e. without MS diagnosis) were selected preferably from healthy people who were referred for normal check-ups. After obtaining their consent, blood samples (5 cc) were taken from subjects of both groups for the *B. pertussis* antibody test. All samples were sent to the hospital laboratory. Laboratory tests were performed by ELISA method using Human Anti-*Bordetella Pertussis*-IgG ELISA kit (manufactured by Abcam Company, United States). Laboratory results were reported as positive or negative cutoffs. Optical absorption higher than 10%, compared to the cut-off, was considered positive.

Also, the nasopharyngeal swab was prepared using Dacron swab and cultured on *Regan Lu medium* (containing “Bordet-Gengou and charcoal agar”). Then, it was incubated in a humid environment for 10-12 days at 36 °C. This culture medium with a shelf-life of 4 to 8 weeks contained charcoal agar with 10% horse blood for better growth of microorganisms as well as 40 mcg of cephalexin in order to inhibit the growth of normal flora of the respiratory tract. In an ideal case, the maximum sensitivity of culture for *B. pertussis* is 60%. Culture media were stored in a wet incubator for 12 days at 35 °C and after 24 h were examined daily. Suspected colonies were primarily determined according to their forms (fine and shiny spots similar to mercury droplets). Then, suspected cultures were fixed, and after gram staining using counterstain “O” and observing gram-negative Coccobacillus, further experiments were performed using Difco TM *B. pertussis* Antiserum (BD company; USA) and slide agglutination tests technique in order to differentiate *B. pertussis* from *Bordetella para-pertussis* [21]. All tests, including antibody and culture, were performed in Sina Hospital.

In order to perform real-time PCR, another swab was prepared for DNA extraction using a high pure PCR template preparation kit (made by ROCH Company; Germany). Then, the extracted DNA was used for real-time PCR using primers mentioned in the Table 1, after performing quantitative and qualitative tests. Molecular analyses were performed in the microbiology laboratory of the medical school.

**Table 1:** Characteristics of primers used for examining the genes

Primers	Sequences (5′ → 3′)	Target	Size (bp)	***T***_**m**_ (°C)
F1-BP	TCCGAACCGGATTTGAGAAA	IS*481*	60	78.5
R1-BP	CCGGGCTCCTTGAGTGAA			
F1-BpP	ATGCTGGATCGCAAGTTGATG	IS*1001*	81	85.8
R2-BpP	TGGGTCTTCGGGCCATT			
F1-16S-TQM	CGTGTCGTGAGATGTTGGGTTA	16S rDNA	60	78.5
R1-16S-TQM	GACGTCATCCCCACCTTCCT			

In this study, specific primers were used. The blast was performed to select primers, and the accuracy of the primers sequence and their specificity for the target gene was confirmed.

### Preparation and dilatation of primers

The synthesized primers were prepared as lyophilized from Bioneer by Pishgam Company. According to the guideline published by the producer, the primers were diluted with a certain sterile distilled water (analysis sheet for each primer). Afterward, in order to be used in PCR reaction, each primer was diluted 10 times. So that 10 μl of primer was mixed with 90 μl of sterile distilled water using a 0.2 ml Micro Tube, which yielded a 10 Piko Molar concentration.

### Real-time PCR and MCA to measure the sensitivity of primers

Applied Biosystem Real-Time PCR and Fermentas Master Mix Kit (Cat No. K0221) were used to perform real-time PCR reactions [22]. In this reaction, for each sample, the materials inside the final mix were added as the reaction material table, 1 μl of the extracted DNA was added to each microtube. Then, it was spin for 15 s and put into the device according to the Table 1, and the program was run. The temperature cycle used in this device was planned for three stages. The first stage, which led to DNA denaturation and contained activation of the polymerase enzyme, was performed at 95 °C for 10 min. The second stage contained a DNA proliferation reaction at 95 °C for 15 s, and a temperature of 58 °C continued for 1 min for 40 cycles. The final stage was performed to draw the melting curve, which is necessary for differentiation of the required genes, at 95 °C for 15 s, 58 °C for 1 min, and 95 °C for 15 s.

Data were analyzed using SPSS. Descriptive data were displayed as tables, diagrams, central indices, and dispersion. For data analysis, the mean *B. pertussis* colonization in MS patients and control group, if the data were distributed normally, was compared using the chi-squared test, Independent Sample t-test, and the Mann-Whitney test was applied for data that were not distributed normally. Statistical significance was considered when *p*-value< 0.05.

### Ethical considerations

The current study is approved by the Ethics Committee of the Hamadan University of Medical Sciences (HUMS) with an ethical code: IR.UMSHA.REC.1396.544.

## Results

The current study aimed to compare the serum level of *B. pertussis* antibody and the level of *Bordetella* nasopharyngeal colonization in 109 patients with MS and 114 healthy individuals. According to Table 2, the case and control groups were matched in terms of vaccination history, age, sex and weight.

**Table 2:** Basic characteristics of subjects, separated by the group

Variable	Groups		*P*.*value*
	Cases (*N* = 109)	Control (*N* = 114)	
Sex	N(%)	N(%)	
Male	17(15.6)	29(25.4)	0.07*
Female	92(84.4)	85(74.6)	
Hx of pertussis vaccination	109(100)	114(100)	^*^0.44
	mean ± Sd	mean ± Sd	
Age (year)	10.5 ± 37.6	9.5 ± 35.8	0.17**
Diseases Duration (year)	9.92 ± 4.97	–	

*Chi-squared test

**Independent Sample t-test

The study’s main results, including colonization rate of the nasopharynx by *B. pertussis* and antibody levels against this bacteria, are listed in Table 3.

**Table 3:** Frequency of *B. pertussis* antibody serum level, colonization rate, and PCR result in patients with MS and control group

Variables	Cases (*N* = 109)	Control (*N* = 114)	*P.value*
Serum *B.pertussis* Ab	37.8%	35.1%	0.74
Nasopharynx culture	0%	0%	–
PCR	0%	0%	–

For 8 (7.3%) patients with MS and 27 (23.7%) subjects of the control group, the nasopharyngeal culture was positive for microbes other than *B. pertussis*. For patients with MS, cultured microbes included gram-negative bacilli (*n* = 1), gram-positive coccus (*n* = 6), and Penicillium fungus (*n* = 1). For subjects of the control group, non-gram negative bacilli (*n* = 3), gram-positive coccus (*n* = 21), and yeast and Penicillium fungus (*n* = 3) were observed.

The frequency of *B. pertussis* antibody in MS patients depends on their age and gender. The mean duration of the disease in patients with MS who were positive and negative for *B. pertussis* antibody was 10.88 ± 5.38 and 9.93 ± 4.96 years, respectively. According to the results of the Mann-Whitney nonparametric test, there was no significant association between serum level of *B. pertussis* in patients with MS and duration of the disease.

## Discussion

In the present study, serum levels of *B. pertussis* antibody in the case and control groups were 37.8 and 35.1%, respectively. All subjects were negative for nasopharyngeal colonization and PCR. There was no significant association between *B. pertussis* antibody titer and age, sex, and duration of the disease. The study by Eslamifar et al. [23] in Iran reported that more than half of children aged 8 months to 6 years were negative for *B. pertussis* antibody. However, there was a direct correlation between *B. pertussis* antibody and age.

In the study by Hashemi et al. [24] about half of the students had positive *B. pertussis* titer, which was not different between males and females. In the present study, the frequency of *B. pertussis* positive antibody in the serum of patients was 37.8%, which is lower than the findings of the aforementioned studies. This discrepancy can be attributed to the gradual decline of serum antibodies overtime after developing the disease or vaccination [25], particularly worth noting that subjects of the present study were older than the abovementioned studies.

In the present study, nasopharyngeal colonization and PCR revealed no case of *B. pertussis*. However, there were cases of gram-positive coccus colonization (*n* = 6), gram-positive bacilli (*n* = 1), and Penicillium (*n* = 1). The hypothesis of the association between infectious agents and the likelihood of developing MS was initially suggested based on the ecological study of Kurtzke et al. [19].

According to the study by Zarkesh-Esfahani et al., infectious agents play a significant role in the etiology of MS. However, no researcher has mentioned the contribution of specific microbial agents in the pathogenesis of MS [7]. Infectious agents may cause secretion of cytokines, Gliosis, and leukocyte infiltration due to inflammation and loss of integrity in CNS tissue [26]. *Chlamydia pneumonia*, HH6, EBV, and Mycoplasma are mentioned as infectious agents that may have a role in MS pathogenesis [11, 26–28]. Antigen similarity between these pathogens and CNS tissue has been suggested as a probable cause for the onset of myelin damage [27, 29]. Fiore et al. [20], in a study on 92 patients with a definite diagnosis of MS, reported that 95.47% of subjects were positive for anti- *B. pertussis* antibodies. Researchers reported that high pathogenic toxin may be directly attached to Nero-Epithelium, causing damage to the CNS.

Considering the effect of subclinical colonization of *B. pertussis* on nasopharynx of vaccinated individuals and the influence of *B. pertussis*, as a potential adjuvant agent with neural antigens, in the induction of neuropathology of autoimmune encephala and development of MS in the animal model, as well as based on epidemiological and biological evidence, Rubin et al. concluded that colonization of *B. pertussis* can be an important cause of MS [18]. However, contrary to the studies by Fiore [20] and Rubin [8], Sen et al. [30], in a study on mice, concluded that administration of pertussis toxin did not affect phenotype induction similar to MS.

## Conclusion

There was no difference between *B. pertussis* antibody titer in MS patients and the healthy control group. None of the participants in both case and control groups presented nasopharyngeal colonization of *B. pertussis*. Therefore, it seems that probably *B. pertussis* has not a role in MS development.

## Data Availability

The datasets used and/or analyzed during the current study available from the corresponding author on reasonable request.

## Abbreviations

MS: Multiple sclerosis
*B. pertussis*: *Bordetella pertussis*
PCR: Polymerase Chain Reaction
CSF: Cerebrospinal fluid
HUMS: Hamadan University of Medical Sciences (HUMS)
EBV: Epstein-Barr virus
CNS: Central nervous system
